# Development, Characterization, and Pharmacodynamic Evaluation of Hydrochlorothiazide Loaded Self-Nanoemulsifying Drug Delivery Systems

**DOI:** 10.1155/2014/274823

**Published:** 2014-12-16

**Authors:** Pankajkumar S. Yadav, Ekta Yadav, Amita Verma, Saima Amin

**Affiliations:** ^1^Department of Pharmaceutical Sciences, Sam Higginbottom Institute of Agriculture, Technology & Sciences (SHIATS), Allahabad 211 007, India; ^2^Department of Pharmaceutics, Faculty of Pharmacy, Hamdard University, New Delhi 110 062, India

## Abstract

The objective of the current work was to develop optimized self-nanoemulsifying drug delivery systems (SNEDDS) and evaluate their *in vitro* and *in vivo* performance. The research comprised various studies which includes solubility studies in various vehicles, pseudoternary phase diagram construction, and preparation and characterization of SNEDDS along with *in vitro* dissolution and *in vivo* pharmacodynamic profiling. Based on dissolution profile, a remarkable increase in rate of dissolution was observed in comparison with plain drug and marketed formulation. Optimized SNEDDS formulation was composed of Capmul MCM (19.17% w/w), Tween 80 (57.5% w/w), Transcutol P (12.7% w/w), and HCT (4.17% w/w). *In vivo* pharmacodynamic evaluation in Wistar rats showed considerable increase in pharmacological effect of HCT by SNEDDS formulation as compared with plain HCT.

## 1. Introduction

Solubility, together with permeability, plays significant role in oral bioavailability of a drug [1]. Many conventional drugs present problems related to low solubility in aqueous medium, resulting in a low absorption rate [2, 3]. Several strategies attempted to increase the solubility of poorly water-soluble drugs, that is, micronization [4, 5], solid dispersion [6–8], complexation [9, 10], and so forth.

Lipid based formulation represents a distinctive and relatively novel solution to delivery of poorly soluble compounds. A lipid dosage form usually consists of one or more drugs dissolved in a blend of lipophilic excipients such as triglycerides, partial glycerides, surfactants, or cosurfactants [11]. Among the lipid based systems, the self-nanoemulsifying drug delivery system (SNEDDS) is a potential technology to improve the rate and extent of absorption of poorly water-soluble drugs [12]. SNEDDS are isotropic mixtures of drug, lipids, and surfactants, usually with one or more hydrophilic cosolvents or cosurfactants [13]. Hydrophobic drugs can be dissolved in these systems, enabling them to be administered as a unit dosage form for peroral administration. When such a system is released in the lumen of the gastrointestinal tract, it disperses to form a fine oil in water emulsion (micro/nano) with mild agitations provided by gastric mobility. This leads to in situ solubilization of drug that can subsequently be absorbed by lymphatic pathways, by passing the hepatic first-pass effect [14].

Hydrochlorothiazide (HCT) is a potent diuretic drug that is practically insoluble in water and has a solubility of only 250 *μ*g/mL in 0.1 N HCl at 25°C [15, 16]. HCT has low toxicity and is widely used in combination with cardiovascular drugs for the treatment of hypertension [17]. Poor water solubility causes possible deviation in the oral bioavailability and, therefore, there is great interest in the development of new drug delivery systems that could enhance the solubility and permeability of HCT and, as a consequence, its bioavailability. According to Biopharmaceutical Classification System (BCS), HCT is classified as class IV drug having low solubility and low permeability [18, 19]. SNEDDS can be utilized to enhance drug solubilization in GIT and it has also an impact on permeability [20–22].

Rapid disintegrating tablet strategy has been experimented for HCT using various carriers [23]. However, at present, no HCT marketed products arising from this approach are available, probably because of the unsatisfactory performance of the studied systems. The main objectives of the present study were to develop an optimized self-emulsifying drug delivery system for HCT and assess its pharmacodynamic effect in terms of diuretic efficacy.

## 2. Materials and Methods

### 2.1. Materials

Hydrochlorothiazide was received as a gift sample from Torrent Pharmaceuticals Ltd., Ahmedabad, India. Maisine 35-1, Transcutol P, and Lauroglycol 90 were generously provided by Gattefosse, France. Cremophor RH 40 and Cremophor EL were received as gift sample from BASF, USA. Captex 355 EP/NF, Captex 300 EP/NF, and Capmul MCM NF were a generous gift from Abitec Corporation, USA. Polyethylene glycol 400 (PEG 400) was purchased from Merck Limited, Mumbai, India. Oleic acid was purchased from S. D. Fine Chemicals Limited, Mumbai. Propylene glycol and Tween 80 were purchased from Thomas Baker Chemicals Limited, Mumbai. Castor oil USP was purchased from Arora Pharmaceuticals Private Limited, New Delhi. Empty hard gelatin capsules were obtained from Associated Capsules Pvt. Ltd, Mumbai. Dialysis Tubing (seamless cellulose tubing, MWCO 12000) was purchased from Sigma Chemical Co., USA. All other chemicals used were of analytical grade.

### 2.2. Solubility Studies

These studies were performed to determine the solubility in individual vehicle (Table 1). Highest solubility showing vehicle was then used for formulation of SNEDDS. Initially the solubility of HCT was determined in oils (i.e., Maisine 35-1, Capmul MCM, Captex 355 EP/NF, Captex 300 EP/NF, oleic acid, and castor oil), surfactant (i.e., Tween 80, Lauroglycol 90, Cremophor RH 40, and Cremophor EL), and cosurfactants (Transcutol P, PEG 400, and Propylene glycol). 2 mL of each vehicle was added in capped vial containing excess of HCT. These vials were stirred on a water bath maintained at 30°C for 48 hours. After attainment of equilibrium each vial was centrifuged at 5000 RPM for 10 min to separate the insoluble drug. Excess of insoluble drug was removed by membrane filter of 0.22 *μ* pore size (Pall Life Sciences, India). Dissolved HCT was quantified by UV-spectrophotometer (UV-2202, Systronics, India) at 270 nm.

### 2.3. Pseudoternary Phase Diagram Studies

**Figure 1:**
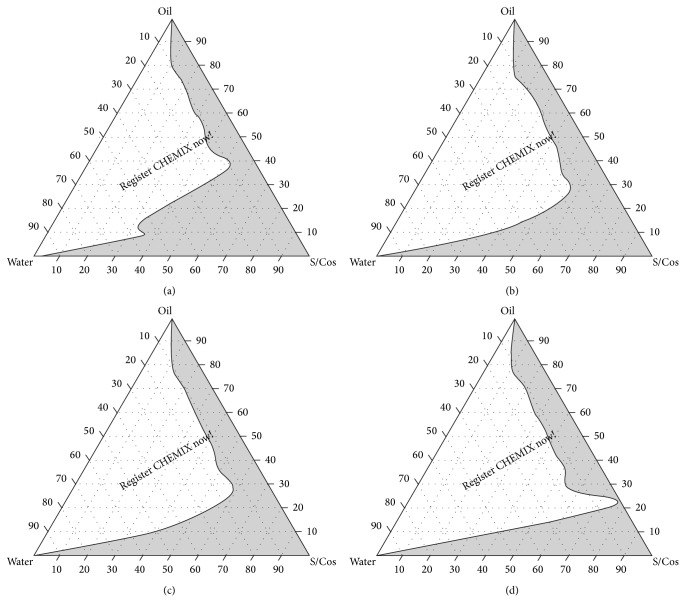
Pseudoternary phase diagrams with the following components: oil = Capmul MCM, surfactant = Tween 80, and cosurfactant = PEG 400. S/Cos ratio of A is 1 : 1, B is 2 : 1, C is 3 : 1, and D is 4 : 1. S/Cos indicates surfactant/cosurfactant. Area in grey shade indicates self-emulsifying region.

**Figure 2:**
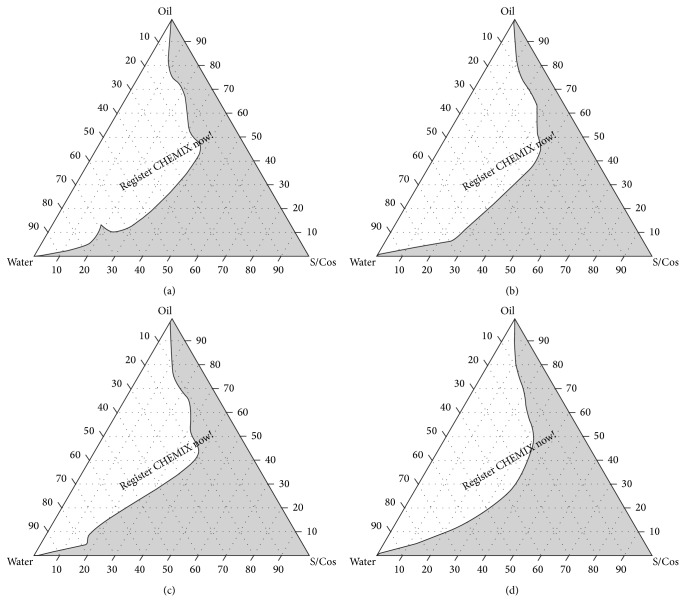
Pseudoternary phase diagrams with following components: oil = Capmul MCM, surfactant = Tween 80, and cosurfactant = Transcutol P. S/Cos ratio of A is 1 : 1, B is 2 : 1, C is 3 : 1, and D is 4 : 1. S/Cos indicates surfactant/cosurfactant. Area in grey shade indicates self-emulsifying region.

Water titration method was used for construction of phase diagram using oil and surfactant/cosurfactant mix (Smix). Based on solubility studies, two sets of Smix (i.e., Tween 80: Transcutol P and PEG 400: Transcutol P) were investigated with Capmul MCM as the oil phase. Surfactant and cosurfactant were added in the ratios of 1 : 1, 2 : 1, 3 : 1, and 4 : 1 for both of the sets. Distilled water was added dropwise to the mixture of certain weight ratios (i.e., 9 : 1, 8 : 2, 7 : 3, 6 : 4, 5 : 5, 4 : 6, 3 : 7, 2 : 8, and 1 : 9) of oil and surfactant/cosurfactant (Smix). Mixtures were stirred using magnetic stirrer. Then each mixture was observed for phase clarity and flowability. Phase diagrams were constructed by using trial version of CHEMIX School 3.50 software (Minnesota, USA) (Figures 1 and 2).

### 2.4. Preparation of SNEDDS Formulations

From the solubility study and ternary phase diagram studies, SNEDDS components were selected for drug incorporation and a series of SNEDDS were prepared (Table 2) with varying ratio of oil to Smix. The series contained Capmul MCM (oil) and Tween 80/Transcutol P (Smix). HCT (4.17% w/w) was loaded into each mixture.

The HCT-SNEDDS was prepared by dissolving drug into Smix in glass vials and accurately weighed oil was added. Components were mixed and heated (45–50°C) to form a homogenous mixture and stored at room temperature till further use.

### 2.5. Characterization and Evaluation of Formulations

#### 2.5.1. Dilution Test

SNEDDS formulation containing 25 mg of drug (1 part) was diluted 10 times with distilled water, 0.1 N HCl, and phosphate buffer of pH 6.8 and observed (Table 3).

#### 2.5.2. Drug Content Determination

Preweighted quantity of HCT containing SNEDDS was dissolved in 25 mL of methanol. HCT content was determined spectrophotometrically (UV-2202, Systronics, India) at 270 nm. Observations are shown in Table 4.

#### 2.5.3. Emulsification Time and Precipitation Assessment

The emulsification time of SNEDDS formulation was assessed on USP II dissolution apparatus (Dolphin, India) (Table 4). Each formulation (600 mg) was added dropwise to 500 mL of distilled water maintained at 37 ± 0.5°C. Gentle agitation was provided by a standard stainless steel dissolution paddle rotating at 50 RPM. Precipitation was evaluated by visual assessment of the resultant emulsion after 24 h. The formulations were then categorized as clear (transparent or transparent with bluish tinge), nonclear (turbid), stable (no precipitation at the end of 24 h), or unstable (showing precipitation within 24 h) (Table 4) [24].

#### 2.5.4. Percentage Transmittance (*ƛ*_max_ 560 nm)

1 mL of SNEDDS formulation was diluted 100 times with distilled water. Percentage transmittance was measured spectrophotometrically (UV-2202, Systronics, India) at 560 nm using distilled water as a blank (Table 4).

#### 2.5.5. Viscosity Determination

SNEDDS (1 mL) was diluted 10 times and 100 times with distilled water in a beaker with constant stirring on a magnetic stirrer. Viscosity of resulting nanoemulsion and initial SNEDDS was determined by using Brookfield R/S plus rheometer (Brookfield Engineering, Middleboro, MA) (Table 4).

#### 2.5.6. Droplet Size Analysis

SNEDDS formulation (600 mg) containing 25 mg of HCT was diluted to 100 mL and mixed gently by inverting the flask. The size of droplet hence formed was measured by using Zetasizer (Malvern Instruments) (Table 4).

#### 2.5.7. Zeta Potential Determination

SNEDDS was diluted 10 times and 100 times with distilled water by constant stirring on a magnetic stirrer. Zeta potential of the resulting emulsion was determined by using Zetasizer (Malvern Instruments) (Table 4).

#### 2.5.8. *In Vitro* Dissolution Studies

*In vitro* dissolution studies were performed to evaluate the dissolution rate of SNEDDS. These studies were carried out in USP type II dissolution test apparatus (Dolphin, India) at 100 RPM in 900 mL of phosphate buffer (pH 6.8). The temperature was maintained at 37 ± 0.5°C.

**Figure 3:**
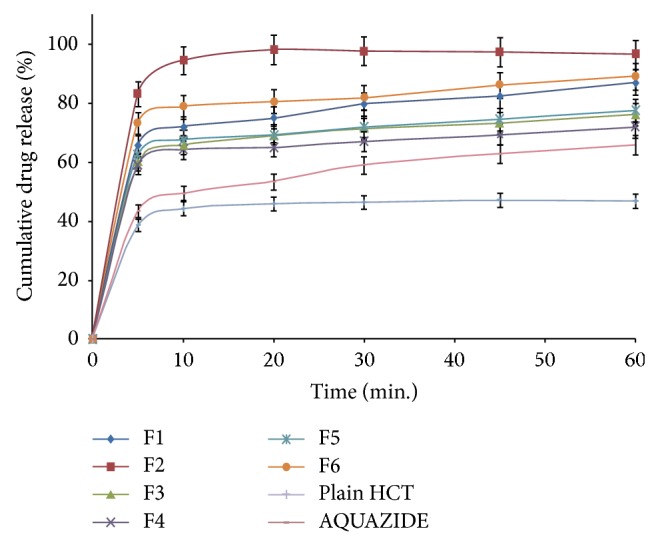
*In vitro* dissolution studies of plain HCT, marketed formulation, and developed SNEDDS, data expressed as mean ± SD (*n* = 3).

600 mg of each SNEDDS formulation (F1–F6) was filled in hard gelatin capsule (size 0) and used for dissolution studies; results were compared with plain HCT and marketed tablet of HCT (AQUAZIDE). 2 mL aliquots were withdrawn at 5, 10, 20, 30, 45, and 60 min intervals and filtered using 0.22 *μ* nylon membranes (Pall Life Sciences, India). The withdrawn samples were diluted suitably and analyzed for the drug content UV spectrophotometrically at 270 nm against phosphate buffer (pH 6.8). An equal volume of the dissolution medium was replaced in the vessel after each withdrawal to maintain the sink condition. Each test was performed in triplicate (*n* = 3), and calculated mean values of cumulative drug release were used while plotting the release curves (Figure 3).

#### 2.5.9. *In Vitro* Diffusion Studies

Permeation of drug through biological membrane was evaluated by* in vitro* diffusion studies carried out by using dialysis technique [25, 26]. One end of pretreated cellulose dialysis tubing (7 cm in length) was tied with thread and 0.3 mL of SNEDDS formulation (equivalent to 10 mg HCT) was placed in it along with 0.7 mL of dialyzing medium (phosphate buffer pH 6.8). The other end of tubing was also tied with thread and was allowed to rotate freely in the dissolution vessel of a USP type II dissolution test apparatus that contained 900 mL dialyzing medium (phosphate buffer pH 6.8) maintained at 37 ± 0.5°C and stirred at 100 RPM. Aliquots were collected periodically and replaced with fresh dissolution medium and analyzed spectrophotometrically at 270 nm for HCT content.

#### 2.5.10. *In Vivo* Studies

*In vivo* study was approved and performed in accordance with the guideline of the animal ethics committee. The rats were housed individually in metabolic cages, controlled conditions of temperature (25°C), and a 12:12 h light/dark cycle. The study was conducted in four groups consisting of three male Wistar rats weighing 250–280 g. Animals were grouped as follows.

 Group I: three rats for plain HCT drug suspension in 0.25% carboxymethyl cellulose (HCT).
 Group II: three rats for optimized SNEDDS formulation (F2) of HCT (SNEDDS).
 Group III: three rats for 0.25% carboxymethyl cellulose (control).
 Group IV: three rats for blank SNEDDS formulation (placebo).

Fifteen hours prior to each experiment food and water were withdrawn. Suspension of HCT (10 mg/kg) and optimized SNEDDS formulation F2 (equivalent to 10 mg of HCT) was administered to animals by gavage performing doses. The four groups of rats were allocated to one of four different treatments as summarized in Table 5. The groups were inverted after providing washout period of 72 hours to each group [27, 28].

Cumulative urine output was recorded at 2, 4, 6, and 8 hours after oral administration of compounds. The urine volume was measured and a urine sample was taken for further analysis. Urinary sodium was determined in a flame photometer (F129, Systronics, India). Results were presented as mean ± S.E.M. (standard error of mean) and were analyzed by two-way analysis of variance followed by Bonferroni post hoc test. A *P* < 0.05 was considered significant.

#### 2.5.11. Stability Studies

Chemical and physical stability of optimized HCT SNEDDS formulation was assessed at 40 ± 2°C/75 ± 5% RH as per ICH Guidelines. SNEDDS equivalent to 25 mg HCT was filled in size “0” hard gelatin capsules, packed in aluminum strips, and stored for three months in stability chamber (CHM 10S, REMI Instruments Ltd., India). Samples were analyzed at 0, 30, 60, and 90 days for clarity, drug content, and time required for 90% drug release (*t*_90%_) (Table 6).

## 3. Results and Discussion

### 3.1. Solubility Studies

Solubility of drug substance is a key criterion for selection of components for developing a SNEDDS formulation. The self-emulsifying formulations consisting of oil, surfactant, cosurfactant, and drug should be a clear and monophasic liquid at ambient temperature. Solubility studies were performed to identify suitable oils, surfactants, and cosurfactants that possess good solubilizing capacity for HCT (Table 1). As HCT was found to have maximum solubility in Capmul MCM, Tween 80, Transcutol P, and PEG 400, further studies were conducted using various combinations of these oils and surfactants to identify the self-emulsifying area. Two sets of Smix and oil in different ratios were used to construct ternary phase diagrams. They were (1) Tween 80 and PEG 400 as Smix and Capmul MCM as oil phase and (2) Tween 80 and Transcutol P as Smix and Capmul MCM as oil phase. For both sets the selected ratios of Smix were 1 : 1, 2 : 1, 3 : 1, and 4 : 1.

### 3.2. Pseudoternary Phase Diagram Studies

Self-nanoemulsifying systems form fine oil-water emulsions with gentle agitation, upon their introduction into aqueous media. Surfactant gets preferentially adsorbed at the interface, reducing the interfacial energy as well as providing a mechanical barrier to coalescence. Figures 1(a), 1(b), 1(c), and 1(d) show ternary phase diagrams of Tween 80-PEG 400 (Smix) and Capmul MCM as oil phase while Figures 2(a), 2(b), 2(c), and 2(d) show ternary phase diagrams of Tween 80-Transcutol P (Smix) and Capmul MCM as oil phase. The area in the shade indicates micro/nanoemulsion region. Wider region indicates better self-emulsifying ability. The cosurfactant helps to achieve prerequisites of emulsion formation and it helps in keeping the surfactant film flexible, fluid, and tightly packed [29]. From the phase diagram studies it can be observed that as the Smix ratio increases the emulsion area decreases. Therefore the cosurfactant plays vital role in the emulsion formation for both of the Smix combinations. The phase study revealed that the emulsion region was more with Tween 80-Transcutol P (Smix) in comparison with Tween 80-PEG 400 combination. Hence the Tween 80-Transcutol P (Smix) was selected for drug loading and further studies.

### 3.3. Dilution Test

The objective of dilution study was to study the degree of emulsification and recrystallization of the drug, if any. Dilution may better mimic conditions in the stomach following oral administration of SNEDDS preconcentrate. Accurate mixture of emulsifier is necessary to form stable nanoemulsion, for the development of SNEDDS formulation, when one part of each SNEDDS formulation was diluted with 10 parts of distilled water, 0.1 HCl, and phosphate buffer (pH 6.8). It was observed that the formulations F1 to F4 were found to be most stable because they do not show any precipitation or phase separation on storage in various dilution media (Table 3).

### 3.4. Characterization and Evaluation of Formulations

#### 3.4.1. Drug Content Determination

Drug content of the SNEDDS formulations is shown in Table 4, which was in the limit (95–102%).

#### 3.4.2. Emulsification Time and Precipitation Assessment

The rate of emulsification is an important parameter for the assessment of the efficiency or spontaneous emulsification of formulation without aid of any external thermal or mechanical energy source. Formulation should disperse completely and quickly when subjected to aqueous dilution under mild agitation of GIT due to peristaltic activity. It has been reported that self-emulsification mechanism involves the erosion of a fine cloud of small droplets from the monolayer around emulsion droplets, rather than progressive reduction in droplet size [30]. The ease of emulsification was suggested to be related to the ease of water penetration into the colloidal or gel phases formed on the surface of the droplet [31, 32]. It was observed that an increase in the proportion of Tween 80 from 33.54 to 57.5% w/w in the composition resulted in increased self-emulsification time from 6 to 18 seconds (Table 4). This might be because of high viscosity imparted by Tween 80 which increases the free surface energy of system thereby increasing the emulsification time with increase in content of surfactant.

Below 44% concentration of surfactant, there was turbid and unstable dispersion (Table 4). This may be due to excess penetration of water into the bulk oil causing massive interfacial disruption and ejection of droplets into the bulk aqueous phase [26].

#### 3.4.3. Percentage Transmittance (*ƛ*_max_ 560 nm)

The percentage transmittance of the six selected optimized formulations was determined. As the value closer to 100% is formulation which is isotropic in nature, optimized formulations of F2-F3 from Smix ratio of 3 : 1 gave maximum percentage transmittance (Table 4). Nanoemulsion formed by SNEDDS in GIT meets with patient acceptabilty but isotropic nature of formulations or percentage transmittance closer to 100% gives an indication of globule size in nanometer range. The droplet size of the emulsion is a crucial factor in self-emulsification performance, because it determines the rate and extent of drug release as well as absorption [33]. Thus, the formulation has the capacity to undergo enhanced absorption and thus the ability to have increased oral bioavailability.

#### 3.4.4. Viscosity Determination

Viscosity of SNEDDS without dilution was found to be in between 302 and 364 cP, which was suitable for filling in hard gelatin capsule without risk of leaking problem. As SNEDDS was diluted 10 and 100 times with water, viscosity of the system was decreased, which indicates that oral administration of SNEDDS formulation will be diluted with the stomach fluid and viscosity will be decreased and therefore absorption from the stomach will be fast (Table 4).

#### 3.4.5. Droplet Size Analysis

The droplet size of the emulsion is an essential factor in self-emulsification performance because it determines the rate and extent of drug release as well as drug absorption. Also, it has been reported that the smaller particle size of the emulsion droplets may lead to more rapid absorption and improve the bioavailability [34]. It is well known that in nanoemulsion systems the addition of surfactants stabilizes and condenses the interfacial film, while the addition of cosurfactant causes the film to expand; thus, the relative proportion of surfactant to cosurfactant has varied effects on the droplet size. From Table 4, it can be seen that formulation F2 has the smallest droplet size of 42.84 nm.

#### 3.4.6. Zeta Potential Determination

**Figure 4:**
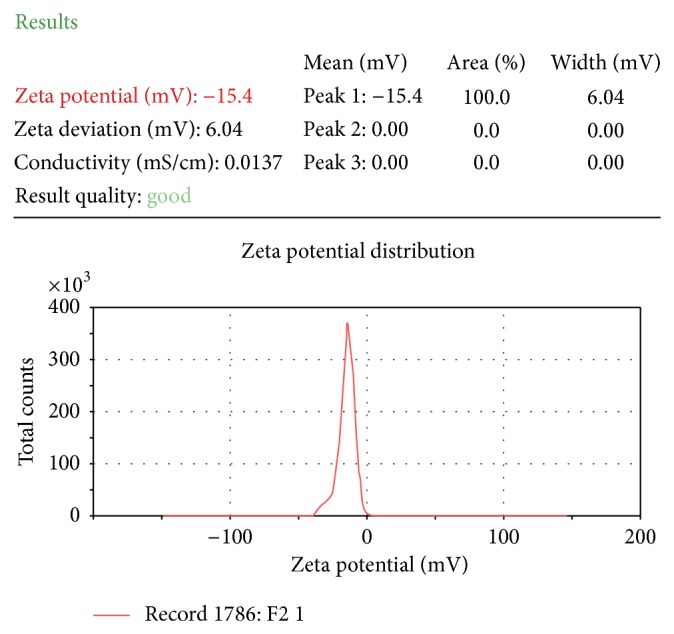
Zeta potential analysis of optimized formulation (F2).

Emulsion droplet polarity is also a very essential factor in characterizing emulsification efficiency [35]. The significance of zeta potential is that its value can be related to the stability of colloidal dispersions. Zeta potential indicates the degree of repulsion between adjacent, similarly charged particles in dispersion. For molecules and particles that are small enough, a high zeta potential will present stability. When the potential is low, attraction exceeds repulsion and the dispersion will break and flocculate. So, colloids with high zeta potential (negative or positive) are electrically stabilized. Negative values of zeta potential of the optimized formulations indicated that the formulations were negatively charged. Formulation F2 was found to be the most stable formulation (Table 4) (Figure 4).

#### 3.4.7. *In Vitro* Dissolution Studies

SNEDDS formulation F2 showed significantly higher drug release as compared to plain HCT and marketed HCT tablet (AQUAZIDE) (Figure 3). F2 showed more than 90% of drug release in 10 minutes while plain HCT and marketed tablet showed 44 and 49%, respectively. Spontaneous formation of nanoemulsion of SNEDDS formulation F2 could be the reason for the faster rate of drug release into the aqueous medium. Dramatic increase in the rate of release of HCT from SNEDDS compared to plain HCT and marketed formulation can be attributed to its quick dispersibility and ability to keep drug in solubilized state. Thus, this greater availability of dissolved HCT from the SNEDDS formulation could lead to higher absorption and higher oral bioavailability.

#### 3.4.8. *In Vitro* Diffusion Studies

**Figure 5:**
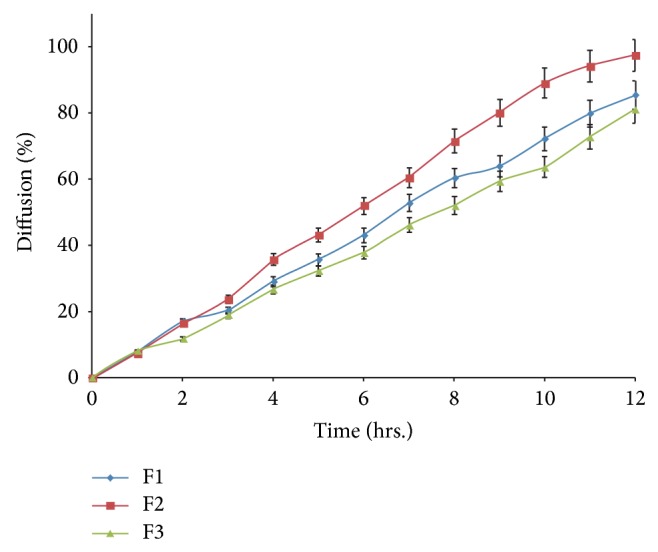
*In vitro* diffusion studies of F2, F4, and F5.

Conventional dissolution testing can only provide a measure of dispersibility of SNEDDS in the dissolution medium of SNEDDS. Alternatively,* in vitro* performance of SNEDDS can be evaluated by drug diffusion studies using the dialysis technique. It is very popular and well documented in many literatures [25, 26]. SNEDDS formulations F1, F2, and F3 were selected for diffusion studies, as these formulations show smaller droplet size among other formulations. Though SNEDDS F6 has smaller droplet size than F3, the former was found to be unstable and turbid on precipitation and clarity test. The release of HCT from these dosage forms was evaluated in phosphate buffer pH 6.8; the release percentage of F2 was significantly higher than that of F1 and F3 (Figure 5). It suggests that HCT dissolved perfectly in SNEDDS form and could be released due to the small droplet size, which permits a faster rate of drug release into aqueous phase. The release rate of HCT from SNEDDS F2 (mean droplet size: 42.84 nm) was faster than SNEDDS F1 and F3 (mean droplet size: 98.48 and 151 nm, resp.). In this study, diffusion profiles of all three formulations (F1, F2, and F3) did not show any differences during initial 1 h; however, at the end of 12 h, formulation F2 showed 97.5% diffusion while F1 and F3 showed 85.5% and 81% diffusion, respectively (Figure 5). Results clearly indicate the effect of mean droplet size on drug diffusion across dialyzing membrane. Hence decreasing the particle size of nanoemulsion could increase the release rate of drug. Therefore, F2 was selected as optimized formulation for* in vivo *studies.

#### 3.4.9. *In Vivo* Studies

**Figure 6:**
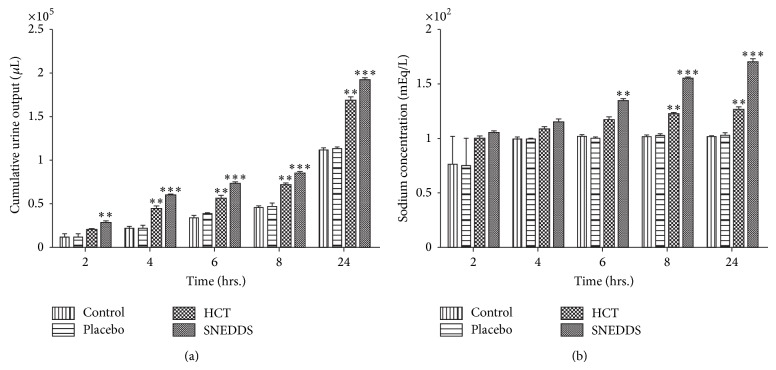
Time course of (a) urine output and (b) sodium output in different groups. Values are reported as mean ± S.E.M. for twelve rats in each group. ^**^Statistically significant from control and placebo group. *P* < 0.05. ^***^Statistically significant from control, placebo, and HCT group. *P* < 0.05.

This study was performed to evaluate the pharmacodynamic potential of an optimized formulation (F2) against plain HCT. Cumulative volumes of excreted urine after oral administration compounds are shown in Figure 6(a). Statistically significant diuretic effect of SNEDDS was observed after 4 hours of administration in comparison with HCT, control, and placebo. This effect was maintained up to 24 hours. The diuretic effect of SNEDDS was significantly different in comparison with HCT, control, and placebo between 4 and 24 hours.

Sodium and chloride ions quantification is one of the best methods to determine diuretic effect of drugs [36, 37]. Values of concentration of sodium in excreted urine are shown in Figure 6(b). SNEDDS group showed significant increase in the amounts of electrolyte in comparison with control, placebo, and HCT groups after 8 hours of administration; however this effect was more but not significant as compared to HCT group up to 6 hours.

Diuretic activity data suggest that SNEDDS formulation increased the pharmacological effect of drug. The higher diuretic activity of the SNEDDS is due to complete dissolution of HCT in SNEDDS, which could have increased absorption. Solubility is a crucial characteristic for increasing the bioavailability of drugs according to the BCS [38]. Moreover, SNEDDS play an important role in improvement of permeability too. Higher permeability may be attributed to Capmul MCM, Tween 80, and Transcutol P as these components have the ability to enhance the permeability [20–22, 39]. However the present work did not deal with the permeation experiments using cell models and this aspect will be developed in future studies.

#### 3.4.10. Stability Studies

Optimized SNEDDS formulation (F2) filled into hard gelatin capsules as the final dosage form. Liquid-filled hard gelatin capsules are prone to leakage and the entire system has a very limited shelf life owing to its liquid characteristics and the possibility of precipitation of the drug from the system. Thus, to evaluate its stability and the integrity of the dosage form, the optimized formulation (F2) was subjected to stability studies. No change in the physical parameters such as homogeneity and clarity was observed during the stability studies. There was no major change in the drug content, drug release (*t*_90%_), and % transmittance (Table 6). It was also observed that the formulation was compatible with the hard gelatin capsule shells. Also, there was no phase separation and drug precipitation was found at the end of three-month stability studies indicating that HCT remained chemically stable in SNEDDS.

## 4. Conclusions

SNEDDS was successfully emerged as appealing approach to improve the bioavailability of HCT. Increased dissolution rate, increased solubility, and ultimately increased pharmacodynamic effect of a poorly water-soluble drug, hydrochlorothiazide, were observed with an optimized SNEDDS formulation consisting of Capmul MCM (19.17% w/w), Tween 80 (57.5% w/w), Transcutol P (12.7% w/w), and HCT (4.17% w/w). The developed formulation showed higher pharmacodynamic potential as compared with plain HCT. Results from stability studies established the stability of the developed formulation.

## Figures and Tables

**Table 1:** Solubility studies of HCT in various vehicles.

Vehicle	Function in SNEDDS	Solubility (mg/mL)
Maisine 35-1	Oil	1.97 ± 0.7
Oleic acid	Oil	2.65 ± 0.85
Capmul MCM	Oil	3.43 ± 0.54
Castor oil	Oil	1.68 ± 0.54
Captex 355 EP/NF	Oil	2.26 ± 0.8
Tween 80	Surfactant	161.46 ± 2.54
Cremophor EL	Surfactant	27.19 ± 1.15
Cremophor RH 40	Surfactant	13.15 ± 1.2
Span 20	Surfactant	1.49 ± 0.73
Lauroglycol 90	Surfactant	1.34 ± 0.74
PEG 400	Cosurfactant	357.14 ± 2.94
Propylene glycol	Cosurfactant	53.60 ± 1.05
Transcutol P	Cosurfactant	288.72 ± 2.56

**Table 2:** Composition of developed formulations.

Formulation codes	Composition (%w/w)			
	Capmul MCM	Tween 80	Transcutol P	Drug
F1	19.17	51.11	25.56	4.17
F2	19.17	57.50	19.17	4.17
F3	28.75	50.31	16.77	4.17
F4	28.75	44.72	22.36	4.17
F5	19.17	38.33	38.33	4.17
F6	28.75	33.54	33.54	4.17

**Table 3:** Observation of dilution test.

Formulation	Distilled water	0.1 N HCl	Phosphate buffer pH 6.8
F1	Stable up to 6 hr	Stable up to 6 hr	Stable up to 6 hr
F2	Stable up to 6 hr	Stable up to 6 hr	Stable up to 6 hr
F3	Stable up to 6 hr	Stable up to 6 hr	Stable up to 6 hr
F4	Stable up to 6 hr	Stable up to 6 hr	Stable up to 6 hr
F5	Unclear within 30 min	Unclear within 30 min	Unclear within 30 min
F6	Unclear within 30 min	Unclear within 30 min	Unclear within 30 min

**Table 4:** Characterization of SNEDDS formulations.

Parameters	F1	F2	F3	F4	F5	F6
Drug content (%)	96.78 ± 1.46	98.88 ± 1.53	98.46 ± 1.07	96.88 ± 1.86	102.34 ± 2.41	98.56 ± 1.97
Self-emulsification time (sec)	15 ± 1	18 ± 2	14 ± 1	9 ± 1	8 ± 1	6 ± 2
Precipitation	Stable	Stable	Stable	Stable	Unstable	Unstable
Clarity	Bluish	Bluish	Bluish	Bluish	Turbid	Turbid
Viscosity (cps)						
0-time dilution	353	364	326	324	321	302
10-time dilution	1.17	1.16	1.15	1.13	1.12	1.08
100-time dilution	0.888	0.883	0.88	0.863	0.857	0.843
% transmittance	55.73	81.43	71.23	57.41	61.54	56.32
Droplet size (nm)	98.48 ± 10.24	42.84 ± 13.78	151 ± 2.67	158.5 ± 15.32	117.5 ± 3.22	95.84 ± 5.42
Zeta potential (mV)	−10.8 ± 0.11	−15.4 ± 0.09	−11.7 ± 0.23	−12.3 ± 0.15	−12 ± 0.18	−13.6 ± 0.21

**Table 5:** Oral administration of pure HCT and optimized SNEDDS formulation F2 compared to control and placebo in Wistar rats.

Treatment	HCT	SNEDDS	Control	Placebo

Period 1	Group 1	Group 2	Group 3	Group 4

Washout period of 72 hours				
Period 2	Group 2	Group 3	Group 4	Group 1

Washout period of 72 hours				
Period 3	Group 3	Group 4	Group 1	Group 2

Washout period of 72 hours				
Period 4	Group 4	Group 1	Group 2	Group 3

**Table 6:** Evaluation data of optimized SNEDDS formulation subjected to stability studies at 40 ± 2°C/75 ± 5% RH.

Sampling points (days)	% drug content	*t*_90% (min⁡)_	% transmittance
0	98.88 ± 1.53	<10	81.43
30	98.31 ± 1.86	<10	82.03
60	97.86 ± 2.32	<10	82.31
90	97.41 ± 2.82	<10	82.28
